# The role of extracellular vesicles in malaria biology and pathogenesis

**DOI:** 10.1186/s12936-017-1891-z

**Published:** 2017-06-09

**Authors:** Natalia Guimaraes Sampaio, Lesley Cheng, Emily M. Eriksson

**Affiliations:** 1 Population Health and Immunity Division, Walter and Eliza Hall Institute of Medical Research, Parkville, VIC Australia; 2 Department of Medical Biology, University of Melbourne, Parkville, VIC Australia; 3 Department of Biochemistry and Genetics, La Trobe Institute for Molecular Science, La Trobe University, Melbourne, VIC Australia; 4 Weatherall Institute of Molecular Medicine, University of Oxford, Oxford, UK

**Keywords:** Extracellular vesicles, Exosomes, Microparticles, Microvesicle, Pathogenesis, *Plasmodium*, Malaria

## Abstract

In the past decade, research on the functions of extracellular vesicles in malaria has expanded dramatically. Investigations into the various vesicle types, from both host and parasite origin, has revealed important roles for extracellular vesicles in disease pathogenesis and susceptibility, as well as cell–cell communication and immune responses. Here, work relating to extracellular vesicles in malaria is reviewed, and the areas that remain unknown and require further investigations are highlighted.

## Background

### Extracellular vesicle biogenesis and function

Extracellular vesicles (EVs) are involved in a wide range of biological processes, and their biogenesis is a highly conserved phenomenon in living organisms [1, 2]. They consist of bi-lipid membrane spheres that are released from cells and contain proteins, lipids, and nucleic acids. These EVs can be taken up by other cells, providing an effective form of cell–cell communication. EVs are generally categorized into exosomes, microvesicles (MV), and apoptotic bodies based on size and biogenesis [3]. Exosomes are the smallest vesicles, ranging in size from 40 to 120 nm, and are made by membrane invagination into endosomes to produce multi-vesicular bodies (MVB). MVBs then fuse with the plasma membrane to release exosomes into the extracellular space. MVs are 50–1000 nm in size, and form by outward budding from the plasma membrane. Apoptotic bodies, which are the largest vesicles (500–2000 nm), result from outward blebbing from the plasma membrane of cells undergoing apoptosis. Microparticles are also often referenced in malaria EV research, and consist of vesicles within the range of 100–1000 nm, which is comparable to the size of MVs, but might differ in the origin of the vesicles as microparticles can also form from blebbing of stressed or apoptotic cells [4]. In this review, microparticles are referred to as MVs.

MVBs were first described in the maturation of reticulocytes into erythrocytes [5], with the term ‘exosomes’ being coined in 1987 by Johnstone et al. [6]. It was first believed that exosomes were used by cells to shed proteins and/or receptors, but a more specialized role for these vesicles was later proposed following reports that they can function in antigen presentation and T cell induction [7]. Exosome formation is now a well-defined mechanism, and occurs via the endosomal sorting complex required for transport (ESCRT) pathway, involving multiple protein complexes [8–10]. However, the kinetics of exosome secretion is still not well understood, although it has been shown to involve Rab GTPases [11]. It is important to note that the majority of reports on the biogenesis of EVs has been studied in mammalian cells, and it is possible that the mechanisms and pathways of EV generation in parasites could differ.

EVs play a myriad of roles in normal physiology and also in diseased states, and have been widely investigated for their role in cancer biology in particular. The protein content of exosomes can be representative of the parent cell, but can also be enriched for certain protein species that reflect both exosome biogenesis and targeting function [1]. Additionally, exosomes can contain mRNA and non-coding RNA, particularly microRNA, providing a mechanism for genetic exchange between cells [12–15]. Valadi et al. first reported the presence of functional RNA in exosomes, and showed that these can be transferred between cells [15]. They demonstrated that exosomes were enriched for particular mRNA and microRNA, and that recipient cells incubated with exosomes derived from donor cells were able to take up and translate the donor exosomal mRNA. Due to EVs being an enriched and protected source of microRNA in bodily fluids, they are now being researched as potential biomarkers for various diseases [16–18].

Recently a mechanism for the specific loading of microRNA into exosomes has been uncovered [19]. Sequence motifs in microRNA can be recognized by heterogeneous nuclear ribonucleoprotein A2B1 (hnRNPA2B1), which guides microRNA for loading into exosomes. Sumoylation is also an important element in this process, as it controls the binding of hnRNPA2B1 to microRNA [20]. This work further demonstrates the specialized role of exosomes in cell–cell communication.

### Extracellular vesicles in infectious disease

It is known that EVs can be released from pathogens (viral, bacterial, and parasitic) as well as from infected cells, and that these EVs are potentially relevant to the infection process and to the immune response to infection [21]. *Mycobacterium tuberculosis*, for example, has been extensively studied in the context of exosomes. Exosomes isolated from *M. tuberculosis*-infected macrophages contain mycobacterial components [22, 23], such as lipoproteins and lipoglycans, and can stimulate TLR2 in uninfected target macrophages [24, 25]. Detailed studies have shown that these immunostimulatory exosomes are likely to be directly of bacterial origin, rather than being host cell-derived exosomes with bacterial components incorporated into them [24]. Host cells of several parasites have been shown to release EVs in response to infection or parasite stimulus, including *Toxoplasma, Trypanosoma, Leishmania*, and *Trichomonas*, and these exosomes can in turn affect the host immune responses. For example, exosomes from *Toxoplasma gondii*-infected macrophages are pro-inflammatory [23], and DCs pulsed with *T. gondii* antigens produced exosomes that contained the parasite antigens [26]. These exosomes were then effectively used to immunize naïve mice against *T. gondii* infection, highlighting the potential of EVs as vehicles for vaccine delivery [26].

In addition to reports that EVs from infected cells are pro-inflammatory [22, 25, 26], there are several studies showing that parasite-derived vesicles can have modulatory effects on the host. Silverman et al. reported that exosomes from *Leishmania* modulated the macrophage/DC response to infection [27]. Specifically, when cells were pre-treated with *Leishmania*-derived exosomes followed by infection with *Leishmania* parasites, there was a reduction in release of pro-inflammatory cytokines IL-8, IL-12 and TNF, and an increase in the anti-inflammatory cytokine IL-10, compared to the untreated cells. The authors further demonstrated that these exosomes were immunosuppressive in vivo, and that HSP100 was involved in the loading of immunosuppressive-specific cargo into exosomes. *Trypanosoma cruzi* also utilize EVs as a method to evade host immunity, by exploiting host-derived MVs to evade complement-mediated immunity and dampen the host immune response [28]. Furthermore, some parasitic helminths also release exosome-like vesicles [29], and can transfer microRNA to host cells to potentially modulate host innate immunity [30].

Not only do parasites utilize EVs to modulate the host immune response, but they are also used as a mode of parasite–parasite communication. Exosomes secreted by *Trichomonas vaginalis* allow inter-parasite communication to promote parasite adherence, which is an important virulence factor, in addition to being taken up by host cells to modulate the host IL-8 response to infection [31]. Most recently, it was found that *Trypanosoma brucei* EV can transfer virulence factor SRA, and can target both other *T. brucei* parasites and host erythrocytes [32]. Thus, EVs are now being recognized to have important functions in parasitic infections, and a hitherto unappreciated role in parasite virulence and immune evasion.

### Host-derived vesicles in malaria

#### Association of host-derived vesicles with malaria severity

Accumulating evidence suggests that EVs contribute to malaria-associated clinical symptoms, in particular in severe disease (Table 1). Initial studies focused on MVs of host cell origin, largely referred to as microparticles. These host-origin MVs were associated with malaria through their role in cerebral pathogenesis, as increased levels of endothelial-derived MVs were present in patients with severe cerebral malaria [33]. The role of MVs in malaria was further dissected using mouse models of severe disease. ABCA1 knock-out mice, which have reduced ability to produce MVs, were protected from cerebral malaria [34]. In control mice, MVs of host platelet, monocyte and endothelial cell origins were released during infection, but this was significantly reduced in ABCA1 knock-outs. In particular, there were reduced inflammatory hallmarks such as high serum TNF and platelet and leukocyte sequestration in the brain. This in vivo work was in agreement with the association of MVs with parasite cytoadherence and their likely role in severe disease caused by parasite sequestration, particularly in cerebral malaria [35]. The importance of ABCA1 in MV-related malaria pathogenesis was further supported by findings from a field study of human patients infected with *Plasmodium falciparum*, where MV levels in patients with severe and uncomplicated malaria were tested for association with their ABCA1 promoter haplotypes [36]. The authors reported that MV release increased during malaria infection, MV levels positively correlated to disease severity, and ABCA1 promotor genotypes were associated with susceptibility to severe malaria. These studies demonstrate that host-derived MVs were an important factor contributing to severe malaria.

**Table 1:** Summary of reports investigating EVs in malaria infection

Vesicle type	Vesicle size	Vesicle isolation method	Cell origin	Study type	Species	Key findings	Report
Host-derived vesicles							
MV	NA	Supernatant from 13,000×*g* centrifugation	Endothelial	Human field study	*P. falciparum*	MVs present in infected individuals.	[33]
MV	NA	Supernatant from 13,000×*g* centrifugation	Endothelial, platelet, erythrocyte	Human field study	*P. falciparum*	MVs in infected individuals are associated with severe malaria and TNF levels.	[38]
MV	NA	Pellet from 100,000×*g* centrifugation	Endothelial, platelet, erythrocyte	Human field study	*P. falciparum*	MVs in infected individuals are associated with severe malaria and ACBA1 gene polymorphisms.	[36]
MV	NA	Supernatant from 13,000×*g* centrifugation	Endothelial, platelet, leukocyte, erythrocyte	Human field study	*P. falciparum*	MVs in infected individuals are associated with cerebral malaria only.	[37]
MV	NA	Pellet from 20,800×*g* centrifugation	Platelet	In vitro	*P. falciparum*	Platelet MVs are involved in iRBC cytoadhesion.	[40]
MV	NA	Pellet from 14,000×*g* centrifugation	Platelet, leukocyte, erythrocyte	Human field study	*P. vivax*	MVs in infected individuals are associated with malaria disease severity.	[42]
MV	NA	Supernatant from 13,000×*g* centrifugation or pellet from 20,000×*g* centrifugation	Endothelial, platelet, monocyte	In vivo	*P. berghei* ANKA	MVs involved in cerebral malaria.	[34]
MV	NA	Pellet from 18,000×*g* centrifugation	Endothelial, platelet, erythrocyte	In vivo and in vitro	*P. berghei*	MVs localize to brain during infection, and can directly induce pathology.	[41]
MV	100–1000 nm	Pellet from 18,000×*g* centrifugation	NA	In vivo	*P. berghei* ANKA	Proteomic characterization of MVs in cerebral malaria.	[4]
Parasite-derived vesicles							
MV	NA	13,000×*g* centrifugation	iRBC	Human field study and in vitro	*P. falciparum, P. vivax, P. malariae*	MVs released from iRBC during active infection.	[48]
MV	100–400 nm	100,000×*g* centrifugation on sucrose cushion	iRBC	In vitro	*P. falciparum*	MVs released from iRBC contain parasite protein and RNA, are immunostimulatory, and induce gametocytogenesis.	[49]
NA	NA	100,000×*g* centrifugation on sucrose cushion	iRBC	In vitro	*P. falciparum*	EVs from iRBC contain functional microRNA that are endocytosed by human endothelial cells and affect barrier properties.	[51]
Exo	70–120 nm	100,000×*g* centrifugation on Optiprep gradient	iRBC	In vitro	*P. falciparum*	Exosomes used for intra-parasitic communication, and induce gametocytogenesis.	[50]
MV	150–250 nm	Pellet from 14,000×*g* centrifugation	iRBC	In vivo	*P. berghei* ANKA	MVs from parasites are pro-inflammatory and stimulate TLR pathways.	[46]
Exo	40–80 nm	Pellet from 100,000×*g* centrifugation, or 100,000×*g* sucrose cushion	iRBC	In vivo	*P. yoelii*	iRBC release exosomes with parasite antigens; exosomes can be used to immunize naïve mice.	[47]

*MV* microvesicle, *Exo* exosome or exosome-like vesicle, *NA* information not available or provided

Recently, a better understanding of the content of host-derived MVs during severe malaria was achieved based on proteomic analysis of MVs from mice with cerebral malaria [4]. There were significant changes in the protein content of MVs from *Plasmodium berghei* (strain ANKA)-infected mice compared to naïve mice, and network analyses indicated that these proteins were actively involved in cerebral malaria pathogenesis. Specifically, TNF and TGFβ1 were predicted to regulate the cerebral malaria-associated proteins in MVs. These findings are in agreement with the association of MVs with cerebral malaria and the involvement of TNF in this disease [4]. However, the field is still lacking in studies on the nucleic acid content of host-derived EVs in malaria. Transcriptomic investigations are needed to further determine if and how host-derived EVs might modulate pathogenesis in malaria, and contribute to disease severity.

Several field studies have reported increased levels of MVs during active *Plasmodium* infection, analysed by flow cytometric analysis of surface stained vesicles, which returned to normal after resolution of infection. However, there have been some differences in reported MV origin, and occurrence during severe disease (Table 1). In *P. falciparum*-infected patients in Cameroon, there was a significant increase in circulating MVs in patients with cerebral malaria, but not in non-cerebral severe or uncomplicated malaria, compared to controls [37]. Platelet-derived EVs in particular were positively associated with severity of cerebral symptoms [37]. Similar results were reported from a field study in India, where MVs originating from platelets, erythrocytes and endothelial cells were increased during infection [38]. In this case, however, the authors reported increased MV levels in cerebral and non-cerebral severe malaria, but not in uncomplicated malaria. Furthermore, high MV levels also correlated with high serum TNF, and levels of MVs and TNF both returned to normal ranges after resolution of infection.

In addition to endothelial cells playing a central role in *P. falciparum* cytoadherence and disease severity, platelet involvement in cerebral malaria is also well established [39]. In vitro, platelet MVs can bind to *P. falciparum* infected red blood cells (iRBC) in a PfEMP1-dependent manner, transfer platelet antigens to iRBC, and induce iRBC cytoadherence to endothelial cells [40]. This has provided mechanistic insights into the involvement of MVs in cerebral malaria, suggesting that EVs can promote cerebral pathology by stimulating iRBC cytoadhesion in the brain. A recent study has investigated the fate and effect of MVs in *P. berghei*-infected mice [41]. They found that when MVs isolated from infected mice were transferred to recipient mice, these MVs localized to cerebral microvessels in infected recipient mice, but not in uninfected recipient mice. Furthermore, transfer of MVs from TNF-stimulated endothelial cells induced brain histopathology similar to cerebral malaria, indicating MVs might be active contributors to the pathologies associated with severe malaria. Notably MV localization to the brain only occurred when the recipient mice were also infected with *P. berghei*, suggesting that the adhesion of these MVs to host organs requires iRBC presence/interaction.

Although most reports have focused on EVs in *P. falciparum,* a study of *Plasmodium vivax* infected individuals in Brazil showed that active infection was also associated with increased MV release, but these MVs originated from platelets, leukocytes and erythrocytes, and not from endothelial cells [42]. Platelet-derived EVs in particular were correlated with high fever, suggesting these host-derived EVs might play a central role in the inflammatory symptoms of *P. vivax* infection [42]. It is interesting to note that endothelial-derived MVs were consistently found to be increased with *P. falciparum* infection (Table 1), which is a cytoadhering parasite, but not with *P. vivax*, which does not cytoadhere. The presence of endothelial cell MVs in *P. falciparum* infection likely reflects the central involvement of this cell type in development of cytoadherent-dependent severe disease symptoms.

Placental malaria is a complication of infection caused by accumulation of iRBC in the placental intervillous space in pregnant women [43], mediated by parasite cytoadherence. A recent study investigated potential links between host-derived MVs and placental malaria [44]. In contrast to severe malarial anaemia or cerebral malaria, there were no changes in total MV or placental trophoblast-specific MV release in women with placental malaria compared to uninfected, indicating that although this severe disease involves parasite sequestration, host-derived MVs are not involved in this specific pathological process during pregnancy-associated malaria. However, there was an overexpression of microRNA miR-517c in MVs from malaria-infected women. miR-517 has been implicated in regulating trophoblast and placental function [45], suggesting that MVs could have applications as biomarkers in placental malaria, and that further research is warranted.

#### Methodological variations in EV research could cause discrepancies

Despite several published reports, there has not been extensive characterization of host-derived MVs from humans infected with malaria. These experiments would be challenging given the high heterogeneity of human samples, but the field would benefit from a more thorough descriptive analysis of vesicles size and composition in these sample types. Furthermore, as shown in Table 1, there are discrepancies in the methodology used to isolate MVs, e.g. some studies investigate the supernatant from 13,000×*g* centrifugation [37, 38], whereas others investigate the pellets from higher centrifugation speeds [36, 40, 41]. These differing methods of sample processing will likely result in varying compositions of the materials being investigated, and cause differing results. Furthermore, many reports did not analyse the morphology of the vesicles being studied, in particular their size, making it difficult to determine exactly what types of vesicles were investigated. Therefore, a consensus on sample preparation is required to ensure that the same vesicle types are being investigated between groups.

Despite these discrepancies, the findings of these studies collectively demonstrate that MVs are specifically released by host cells during malaria infection, and that these MVs mediate a variety of pathological effects. Their correlation with increased inflammation and iRBC cytoadherence indicates that MVs might be important causative agents of severe cerebral pathogenesis in particular. Currently, it remains difficult to determine whether malaria-associated inflammation causes increased MV release, and the MVs themselves are key mediators of pathogenesis; or if the malaria-associated pathology occurs first and the MV release is a secondary outcome of activated cells. Further work, in particular more detailed proteomic and genomic analyses of human-derived MV content, is still required to tease out effects that might be causative versus correlated between host-derived MV release and severe malaria.

### *Plasmodium*-derived vesicles

#### *Malaria mouse models provide insights into* Plasmodium-*derived EVs*

Mouse models of malaria have allowed further understanding of the origins, roles, and effects of EVs in *Plasmodium* infection. Although host-derived EVs have been extensively studied in malarial disease, there have also been investigations into EVs of parasitic origin. A report by Couper et al. showed that MVs from the serum of *P. berghei* ANKA-infected mice had a strong pro-inflammatory effect, stimulating macrophage CD40 surface expression and TNF release in a dose-specific manner [46]. Furthermore, these MVs were mostly of iRBC origin because they contained *P. berghei* antigens. Using a variety of knock-out mice, the authors demonstrated that the stimulatory effects of MVs were not due to a generalized inflamed state, but specific to high density parasitic infection. Furthermore, the stimulatory effect of MVs was found to be dependent on TLR signaling, as there was no response from MyD88-/- and TLR4-/- macrophages, indicating that ligands that stimulate the TLR pathway are present in MVs [46]. However, this study only looked at TNF release and CD40 upregulation as a measure of macrophage activation, and it is likely that other pathways (including other TLRs) are also activated by MVs and induce release of cytokines that were not tested for.

Much of the work into EVs in malaria has focused on MVs, rather than exosomes. However, a study on mice infected with *Plasmodium yoelii* 17X, which preferentially invades reticulocytes, similarly to *P. vivax*, showed that infected reticulocytes released EVs that had exosome-like markers and contained parasite-derived proteins [47]. They also reported that immunization of mice with the iRBC-derived EVs induced iRBC-specific antibodies and protection from lethal infection. Furthermore, this was the first demonstration that exosome-like vesicles, rather than the larger MVs, are released from *Plasmodium*-parasitized cells.

#### *EVs are released by various human*-*infective* Plasmodium *species*

To date, there has been only one study of *Plasmodium*-derived EVs from human field samples. Nantakomol et al. investigated RBC-derived MVs in patients infected with *P. falciparum, P. vivax* and *Plasmodium malariae* [48]. The authors distinguished MVs of iRBC origin from MVs of uninfected RBCs based on the presence of parasite protein RESA, which is inserted on the membrane of parasitized RBC, and showed that iRBC released >tenfold more MVs than uninfected RBC, suggesting an active shedding of vesicles from iRBC. In agreement with previous work, MV numbers increased upon malaria infection, and high MV concentrations correlated with higher parasitaemia and severe disease. This study also investigated *P. falciparum* MV release in vitro, and showed that MV release increased as the parasite matured within the RBC. Additionally, the authors linked induction of MV release to the presence of hemin or parasite products, but this was only shown for uninfected RBC and not with iRBC. This work demonstrated that MVs were released from various human-infective *Plasmodium* species in vivo, and that these contained parasite-derived proteins. However, there are still no reported investigations into what biological functions these *Plasmodium*-derived EVs might have during active infection.

#### Plasmodium falciparum *vesicles in cell*–*cell communication*

The majority of reports on EVs in human malaria investigated host-derived MVs, but two recent studies have looked at the function of parasite-derived rather than host-derived vesicles, and independently reported that *P. falciparum* EVs can mediate cell–cell communication between parasites [49, 50]. Regev-Rudzki et al. utilized genetically modified parasites to demonstrate intra-parasitic exchange of genetic material via vesicles. They co-cultured two different parasite strains, each containing plasmids encoding different fluorescent markers and separate drug resistance genes (for resistance to WR99210 or blasticidin), in the presence of both WR and blasticidin. They found that, despite each strain having resistance exclusively to one drug, parasites were able to survive treatment with both drugs, and the next generation of parasites harbored both drug resistance genes and fluorescent markers. This plasmid transfer was found to occur via extracellular exosome-like vesicles released from the iRBC, and plasmid transfer was observed to occur most efficiently during early ring stage of the asexual life cycle. The authors showed that drug resistance could be transferred to drug-sensitive iRBC by incubation with vesicles purified from the co-culture supernatants. Furthermore, they demonstrated that induction of this vesicle-mediated cell–cell communication also led to increased gametocytogenesis in vitro, suggesting that parasite-derived EVs might provide a means of quorum sensing to trigger gametocytogenesis.

Mantel et al. extensively described *P. falciparum* MV production and composition. In contrast to Regev-Rudzki et al. they investigated vesicles produced by late-stage trophozoites/schizonts that were larger in size. The MVs were profiled by proteomics and Western blotting, and were found to contain parasite-specific proteins, such as SBP1 and RESA, indicating that these MVs are of parasite origin, though the contribution from host cells in this process remains unclear. Similar to Regev-Rudzki et al. the authors also reported that vesicles derived from *P. falciparum* cultures induced gametocytogenesis in recipient cultures. However, it is important to note that these two studies used different methodology to isolate vesicles, and therefore the EVs investigated for each study could represent different subpopulations. Furthermore, the method by which vesicles induced gametocytogenesis was not determined in either study. Moreover, Mantel et al. demonstrated that when monocyte-derived macrophages were stimulated with MVs, they upregulated the expression of IL-1β, IL-6, IL-10 and IL-12, and specifically released IL-10 and TNF, showing that vesicles had an effect on host cells.

Further in vitro studies by Mantel et al. [51] revealed that *P. falciparum* iRBC-derived EVs could affect host endothelial cells through transfer of functional microRNA. Specifically, the authors showed that EVs from iRBC contain host-derived microRNA and Argonaute2 protein, a member of the RNA-induced silencing complex, and that these EVs can be taken up by human endothelial cells. It was further demonstrated that EV-delivered microRNAs reduced expression levels of target proteins and affected the barrier function of endothelial cells. This work suggests a potential targeted effect of EVs on host cell function, which could have important implications for the understanding of clinical disease. Still, the role of parasite-derived vesicles in *P. falciparum* biology and host-related functions in vivo remains largely undetermined.

### Potential of extracellular vesicles in malaria vaccine development

The development of an effective vaccine against malaria is of major global interest, but due to the complexity of the *Plasmodium* parasite life cycle, and large surface protein redundancy, this remains a challenge. Although many candidate antigens have been evaluated as potential anti-malarial vaccines, success has been limited [52]. The RTS,S vaccine, the only currently approved vaccine against malaria, showed limited effectiveness in a recent Phase III clinical trial [53]. Nevertheless, several efforts are still underway to develop an effective and long-lasting anti-malarial vaccine, and new methods of delivery. There is considerable interest in utilizing EVs to enhance vaccine delivery [54], and new technologies for mass production of EVs, such as with exosome-mimetic nanovesicles, could provide a viable therapeutic approach to anti-malarial vaccine development [55–57].

In vivo studies with *P. yoelii* [47], which used iRBC-derived EVs to successfully immunize mice against lethal infection, showed the immunization potential of parasite-derived exosomes. However, most of the research has focused on use of synthetic microparticles/microspheres, such as poly-lactic-*co*-glycolic acid (PLGA), as vehicles for a malaria vaccine. Administration of PLGA vesicles loaded with *P. vivax* antigens [e.g. merozoite surface protein-1 (MSP-1), apical membrane antigen-1 (AMA-1), or circumsporozoite protein (CSP)] through an intranasal mucosal route showed improved humoral and cell-mediated immune responses compared to standard adjuvant vaccination, highlighting PLGA vesicles as an improved immunization strategy [58, 59]. Other strategies consist of using PLGA microparticles to deliver *Plasmodium* antigen-encoding plasmid DNA to antigen-presenting cells to elicit an immunizing response [60], or conjugating these microparticles with additional strong adjuvant molecules to increase vaccine immunogenicity [61].

Transmission-blocking vaccines that target sexual stage parasite or mosquito midgut antigens are also being tested as an anti-malarial vaccine, but due to the absence of natural antigen presentation in the human host, these strategies lack natural boosting and thus have limited efficacy [62]. However, use of biodegradable microparticle packaging of antigens, which allow controlled slow release of the antigen, can elicit long-lasting functional antibody responses and make the vaccine more effective [62]. Further manipulation of lipid vesicles with improved adjuvants can advance this vaccination strategy to allow greater humoral immunity and potency of the vaccines [63]. Thus, a combination vaccine containing leading candidate antigens, delivered via microparticles/EVs or mimetic nanovesicles, have the potential to be the best strategy for vaccination against malaria [1, 63, 64].

### Future directions of EV research in malaria

The discovery that parasite-derived exosomes can transfer drug resistance genes [50] has revealed hitherto unexpected potential mechanisms for horizontal gene transfer in wild parasite populations, and might be of particular relevance in the current state of emerging drug resistance [65]. Furthermore, EV (synthetic or of biological origin) might serve as effective vaccine delivery mechanisms for malaria. Vaccination against *Plasmodium* continues to be a major challenge in the field, despite decades of research, but results from the delivery of leading vaccine candidates in EV/microparticles is promising, and encourages further investigation.

There have been several studies in other parasites demonstrating sophisticated host manipulation exerted by the parasite via EVs [27, 31]. Reports that human immune cells can detect and respond to *Plasmodium* MVs as an immunostimulatory agent [49], and that EVs from iRBC can contain functional microRNA that affect endothelial cells [51] suggest that *Plasmodium* could actively manipulate the host via vesicles, though further investigations are still needed. In particular, the potential that *Plasmodium* might utilize EVs to modulate the host immune system is of significant interest. Alternatively, vesicles could also target host RBC and alter or prepare them for parasite invasion, providing a more permissive environment for successful infection. This remains to be determined, and is an area that requires further investigations.

## Conclusions

**Fig. 1:**
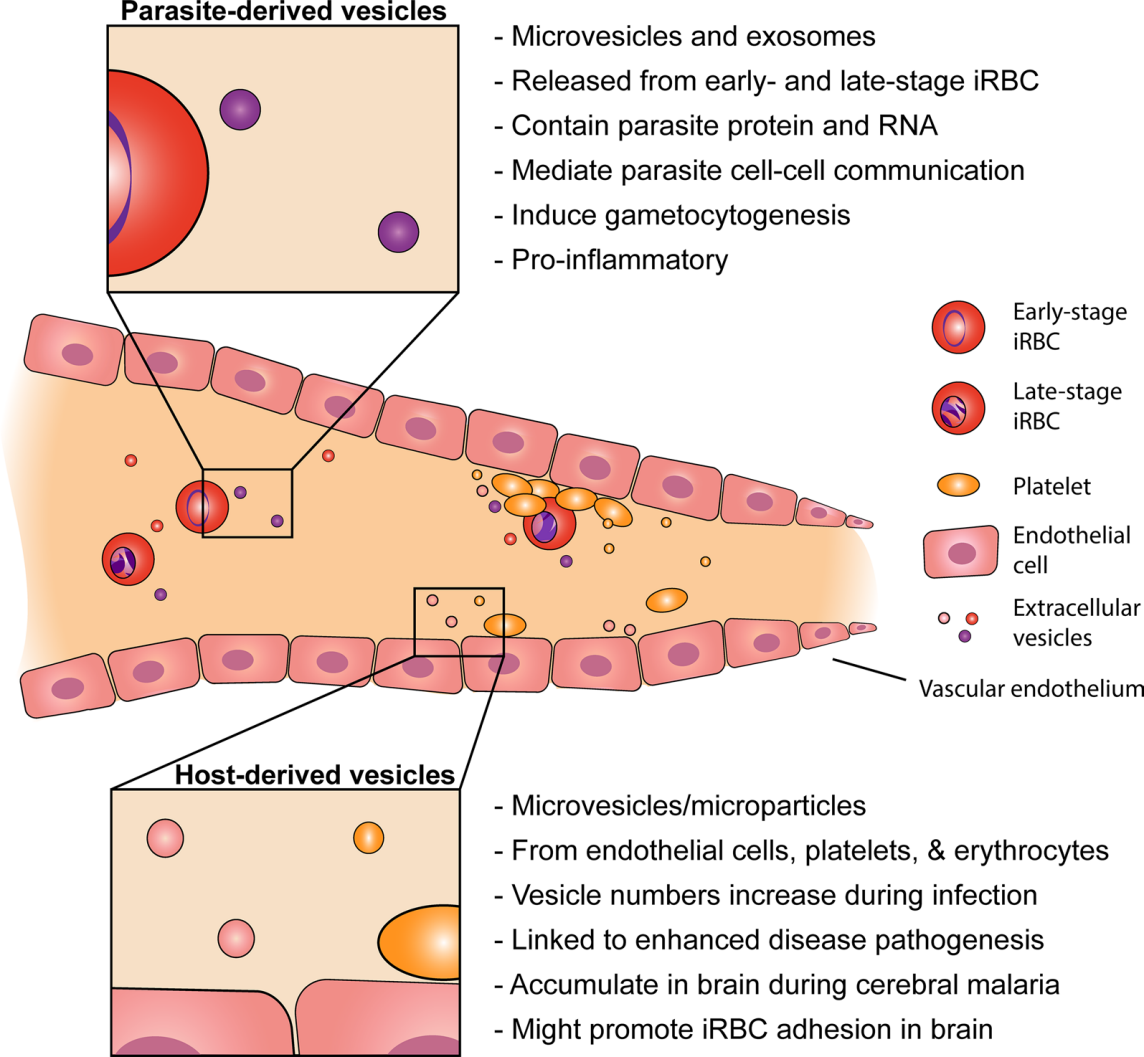
Extracellular vesicle involvement in malaria disease. Extracellular vesicles from both host and parasite origin are released during malaria infection. Exosomes and microvesicles from iRBC have been described, and found to contain parasite material, be pro-inflammatory, induce gametocytogenesis, and mediate cell–cell communication between parasites. Host-derived microvesicles/microparticles released from endothelial cells, platelets, monocytes and erythrocytes have been shown to be involved in malaria disease severity and pathology, in particular in cerebral malaria. Host microvesicles likely contribute to the iRBC cytoadhesion to the vascular endothelium

There has been considerable research on the roles of EVs, originating from either the host or the parasite, in malaria biology and pathogenesis. Host-derived MVs have been implicated in severe malaria pathogenesis. These MVs are released from various cell types, but those of endothelial and platelet origin have been most commonly found to mediate pathology, including induction of cytoadhesion of iRBC, and effects on microvasculature in the brain. EVs of parasitic origin have also been investigated in vivo and in vitro, in both human samples and mouse models. These EVs have been shown to contain parasite material, induce pro-inflammatory responses, transfer functional microRNA, and mediate cell–cell communication between parasites in vitro, allowing a mechanism of nucleic acid exchange as well as quorum sensing (Fig. 1). Additionally, *Plasmodium* exosomes can induce antibody-mediated immune protection in mouse models, and have the potential to provide more effective vaccine delivery strategies. Nevertheless, it is still unclear what roles these EVs might play during active infection in humans, and what global modulatory effects they have on the host, thus additional research in this field is still warranted.

## Abbreviations

EV: extracellular vesicle
MV: microvesicle
iRBC: infected red blood cell
uRBC: uninfected red blood cell
MVB: multi-vesicular body
